# A Fine-Scale Phylogenetic Analysis of Free-Living *Burkholderia* Species in Sugarcane Field Soil

**DOI:** 10.1264/jsme2.ME14122

**Published:** 2014-11-20

**Authors:** Kanako Tago, Hideomi Itoh, Yoshitomo Kikuchi, Tomoyuki Hori, Yuya Sato, Atsushi Nagayama, Takashi Okubo, Ronald Navarro, Tomo Aoyagi, Kentaro Hayashi, Masahito Hayatsu

**Affiliations:** 1 Environmental Biofunction Division, National Institute for Agro–Environmental Sciences, 3–1–3 Kannondai, Tsukuba, Ibaraki 305–8604, Japan; 2 Bioproduction Research Institute, National Institute of Advanced Industrial Science and Technology (AIST) Hokkaido, 2–17–2–1 Tsukisamu-higashi, Toyohira-ku, Sapporo, Hokkaido 062–8517, Japan; 3 Research Institute for Environmental Management Technology, AIST, Tsukuba, Ibaraki 305–8569, Japan; 4 Okinawa Prefectural Agricultural Research Center, Itoman, Okinawa 901–0336, Japan

**Keywords:** *Burkholderia*, Illumina sequencing, sugarcane field

## Abstract

The diversity and abundance of *Burkholderia* species in sugarcane field soils were investigated by a 16S rRNA gene-based approach using genus-specific primers. A total of 365,721 sequences generated by the Illumina MiSeq platform were assigned to the genus *Burkholderia*. Nearly 58% of these sequences were placed in a previously defined cluster, including stinkbug symbionts. Quantitative PCR analysis revealed a consistent number of 16S rRNA gene copies for *Burkholderia* species (10^7^ g^−1^ soil) across the sampled fields. C/N, pH, and nitrate concentrations were important factors shaping the *Burkholderia* community structure; however, their impacts were not significant considering the overall genus size.

The genus *Burkholderia* currently comprises more than 60 species. There are two main clusters within the genus, the *Burkholderia cepacia* complex (BCC), which consists of species known to cause diseases in plants and animals, and the plant-associated beneficial and environmental (PBE) group (7, 21). Some species in the PBE group are beneficial to their plant hosts (6, 21) and other species can degrade xenobiotics (8). A third cluster composed of bacterial symbionts associated with the stinkbug *Riptortus pedestris* and its relatives has recently been proposed as “the stinkbug-associated beneficial and environmental (SBE) group” (9, 10, 11).

Recent field surveys detected *Cavelerius saccharivorus*, which hosts the SBE or PBE group, in sugarcane fields on a Japanese island (9). *C. saccharivorus* acquires their symbionts by horizontal (environmental) or vertical (transovarial) transmission (9). Horizontally acquired symbionts are assumed to be derived from a genetically diverse free-living population, *e.g.* in agricultural soil, which is considered a reservoir for *Burkholderia* species (5). However, few studies have examined the diversity and abundance of *Burkholderia* stinkbug symbionts and their relatives in soil environments.

High-throughput sequencing methodologies using specific primers are known to effectively provide an insight into the diversity of bacterial groups at a fine scale (1, 2). Therefore, we explored the diversity and abundance of *Burkholderia* species, especially the SBE and PBE groups, in sugarcane field soil using Illumina sequencing and qPCR.

To design a set of specific primers for the detection of *Burkholderia* species, we aligned the 16S rRNA gene sequences of type strains obtained from the GenBank database with those of our collection of strains isolated from agricultural soils (22). The forward primer Bf (5′-TAGCCCTGCGAAAGCCG-3′), from positions 127 to 143 bp with reference to the 16S rRNA sequence of *B. multivorans* LMG13010^T^ (GenBank accession no. Y18703), was modified from BKH143Fw1 (19). The reverse primer Br (5′-GCCAGTCACCAATGCAG-3′), from positions 608 to 624 bp of the *B. multivorans* LMG13010^T^ 16S rRNA sequence, was modified from Burkho4A (18). The PCR fragment contained the v2, 3, and 4 regions of the 16S rRNA gene. The specificity of the primers was evaluated *in silico* using BLAST and the Ribosome Database Project (RDP) II classifier. Genomic DNA from *Burkholderia* type strains and other *β*-Proteobacteria (Table S2) and from bulk soil samples of sugarcane fields were used to confirm primer specificity. The purity of the PCR products was confirmed by electrophoresis on a 1.5% agarose gel, on which a distinct band (*ca.* 463 bp) was obtained from *Burkholderia* type strains as well as soil samples. The PCR products of the soil samples were further subjected to cloning and sequencing by standard techniques, and all the cloned sequences (*n* = 38) were identified by a BLAST search as belonging to *Burkholderia* species (data not shown).

Our sampling site, Minami-Daito Island (25°50′N, 131°14′), is located in the Philippine Sea, 360 km east of Okinawa Island, Japan. Soils were classified as either Lateritic Red soil or Lateritic Yellow soil. Agricultural land, mainly sugarcane fields, covers approximately 60% of the total area (30.57 km^2^) of the island. Soils were sampled from 11 farmers’ sugarcane fields on June 2010 (Fig. S1). The soil samples were collected from furrows at three locations within each field, and sieved through a mesh with a 2 mm pore size. The chemical properties of the different soil samples are listed in Table S1. Soil DNA was extracted from each of the three subsamples (0.4 g) using the Fast DNA SPIN Kit for Soil (Q-Bio, Carlsbad, CA, USA), and further purified with the DNA Clean and Concentrator™ (Zymo Research Corp., Orange, CA, USA).

The purified DNA (10 ng) of each subsample was subjected to PCR amplification of the *Burkholderia* 16S rRNA gene for Illumina sequencing using the *Burkholderia*-specific primers Bf and Br, with barcodes (3). A temperature profile was 30 cycles at 94°C for 30 s, 58°C for 1 min, and 72°C for 30 s. The PCR amplicons (*ca.* 498 bp) were purified with AMPure XP beads (Agencourt Bioscience, Beverley, MA, USA) and gel extraction using the QIAquick Gel Extraction Kit (QIAGEN, Valencia, CA, USA). The quality of the purified amplicons was assessed using the Agilent 2100 Bioanalyzer (Agilent Technologies, Santa Clara, CA, USA). The purified amplicons were quantified using the Quant-iT™ DNA Assay Kit (Life technologies, Carlsbad, CA, USA), and pooled in equal molar concentrations. A DNA library containing the amplicons and internal control PhiX were used for paired-end sequencing using the MiSeq sequencer (Illumina, San Diego, CA, USA) and MiSeq Reagent Kit v3 (Illumina) according to the manufacturer’s instructions. In addition to the processing of raw datasets as described previously (9), sequence reads with length <462 bp or >467 bp, which was outside the length of the reference sequences of *Burkholderia* species (127 sequences, listed in Fig. S2), were discarded using the Mothur program (http://www.mothur.org). The taxonomic assignments for the trimmed sequences were determined by the RDP multiclassifier at a confidence level of 0.50 (23). Phylogenetic analyses were performed using the maximum-likelihood method (17) implemented in MEGA (http://www.megasoftware.net/).

Quantitative PCR was performed with the StepOnePlus™ Real-Time PCR System (Applied Biosystems, Foster City, CA, USA) and SYBR Premix Ex Taq Kit (TaKaRa Bio, Otsu, Japan). The 16S rRNA gene from soil DNA was amplified by PCR using the Bf and Br primers, with a temperature profile of 40 cycles at 94°C for 30 s, 56°C for 30 s, and 72°C for 30 s. The copy number of the 16S rRNA gene was calculated using a standard curve. To construct the standard curve, the PCR amplicons of 16S rRNA gene derived from *B. plantarii* LMG9035^T^, *B. multivorans* LMG13010^T^, and *B. silvatlantica* NBRC106337 were used as reference strains. A mixture containing equal amounts of the three 16S rRNA gene amplicons of the reference strains was serially diluted and subjected to analysis. PCR efficiency and the coefficient of determination (R^2^) for the standard curves were 88.9% and 0.998%, respectively.

A pyrosequencing approach using genus-specific primers has previously been used to clarify the distribution of *Burkholderia* species in soil (1) and plants (2). However, a sufficient number of sequence reads has not been obtained from complex environments, such as soil, because only 8% of sequence reads generated by the existing primer set have been assigned to *Burkholderia* species (1). After filtering and trimming processes according to our strict criteria, 95.8% of the sequence reads of the raw datasets belonged to the genus *Burkholderia*, according to the RDP classification. The remaining 4.2% of the sequences were assigned to genera such as *Azohydromonas* (1.1%) and *Caldimonas* (0.4%) (Table S3). A total of 365,721 reads assigned to *Burkholderia* species were used for further phylogenetic analysis. The average number of sequence reads per field was 33,247, ranging from 12,250 to 88,909 (Table S4). The sequence reads were classified into 95 operational taxonomic units (OTUs), with a similarity cut-off of 99%. Coverage was 100%, and the Chao 1 estimate was the same as the number of OTUs identified in all the libraries (Table S4), confirming that the sequence reads could cover the entire genus of *Burkholderia*. Phylogenetic analysis revealed that the OTUs were widely distributed within the genus (Fig. S2). These results suggested that the sequence reads (*ca.* 463 bp) were of sufficient length to estimate genetic variations in *Burkholderia* at the species level. These results clearly indicated that Illumina sequencing with our specific primers represented a useful tool for analyzing the *Burkholderia* community structure in detail.

We found high taxonomic diversity for the *Burkholderia* community in the sugarcane field soils (Fig. S2). Similar to the clustering of the representative strains reported by Estrada-de los Santos *et al.* (7), the BCC, PBE, and SBE groups represented distinct clusters in the phylogenetic tree, except for part of the PBE group that was distributed in the BCC (Fig. S2). Of the 95 distinct OTUs, 29 were assigned to the SBE group, which accounted for 7.9 to 94.0% of the intrinsic *Burkholderia* community in each soil sample (Fig. 1, grey bars). As observed above, the diversity and abundance of the *Burkholderia* species relative to the stinkbug symbionts was shown to be high in the sugarcane field soils on the island.

In the phylogenetic tree (Fig. S2), the remaining 58 and 8 OTUs were assigned to the PBE group and BCC, respectively. The PBE group represented 6.0 to 90.0% of the *Burkholderia* community (Fig. 1, open bars). Only a small proportion of the BCC sequences were detected in each soil sample, ranging from 0.01 to 2.0% of the total community (Fig. 1, black bars). The N_2_-fixing and endophytic bacteria assigned to *B. phytofirmans*, *B. silvatlantica*, *B. tropica*, *B. unamae*, and the BCC have been isolated from the rhizospheres and plants of field-grown sugarcane in Brazil, Mexico, and South Africa (4, 13–16). The OTUs closely related to these species were also detected in the soil on the island, suggesting that these species were prevalent in sugarcane fields across the sea.

The *Burkholderia* 16S rRNA gene sequence was detected in all soil samples varying from 1.0 × 10^7^ to 2.8 × 10^7^ copies g^−1^ dry soil (Fig. 1, diamonds). The abundance of *Burkholderia* species in the soil samples was nearly consistent with that of previous findings using qPCR analysis targeting 16S rRNA genes (12); however, information regarding the *Burkholderia* gene copy number in the environment is extremely limited. Based on the MiSeq and qPCR data, the average population sizes of the SBE and PBE groups were estimated to be 1.1 × 10^7^ copies g^−1^ dry soil and 8.4 × 10^6^ copies g^−1^ dry soil, respectively. These results provided strong evidence for the sugarcane field soil on the Minami-Daito Island being a rich source for symbionts that have the potential to associate with stinkbugs.

A canonical correlation analysis (CCA) was used to evaluate the link between the community structure and soil chemical characteristics (Fig. 2). C/N, pH, and nitrate concentrations were found to have marked effects on the *Burkholderia* community structure (Monte Carlo test, *P* = 0.002) (Fig. 2). In contrast, the *Burkholderia* copy number was associated with pH at a low significant level (Pearson’s correlation, r = −0.55, *P* = 0.080), but not with C/N (r = 0.36, *P* = 0.97) or nitrate concentrations (r = −0.01, *P* = 0.272). Therefore, those soil characteristics affected the composition of the *Burkholderia* community, but their impacts were small relative to the overall size of the community.

We herein demonstrated that the SBE and PBE groups accounted for an average of 57.6% and 42.1%, respectively, of the total *Burkholderia* community. These values corresponded to an estimated more than 10^6^ copies g^−1^ soil observed in sugarcane fields. Species in the SBE group have rarely been detected in soils at a continental scale using clone library analysis (20). Our Illumina sequencing strategy demonstrated that the sugarcane field soil was a large reservoir for SBE group and such a location may provide these bacteria the opportunity for *Burkholderia* symbionts to encounter stinkbugs. This strategy can be used to determine the distribution of *Burkholderia* species in various environments, such as those of free-living types with those of the interior of host organisms. Furthermore, by comparing those distributions, a question that has been overlooked by classical molecular techniques (*e.g.*, clone libraries) may be resolved.

The Illumina sequencing data were deposited in the MG-RAST database (http://metagenomics.anl.gov/) under the ID: 4577487.3–4577619.3.

## Supplementary Information



## Figures and Tables

**Fig. 1:**
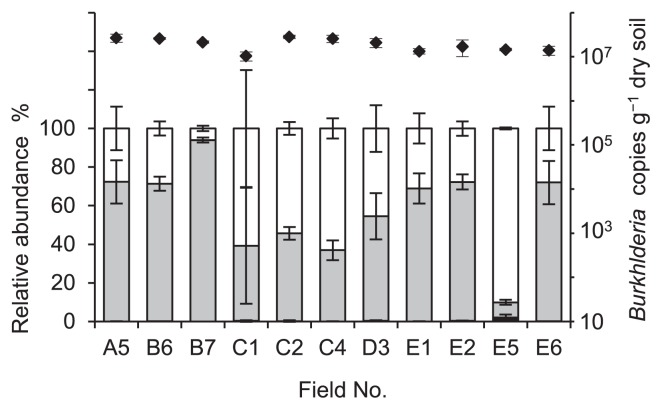
Relative abundances of the SBE group (grey bars), PBE group (open bars), and BCC (black bars), and *Burkholderia* 16S rRNA gene copies (closed diamonds) in the sugarcane field soils.

**Fig. 2:**
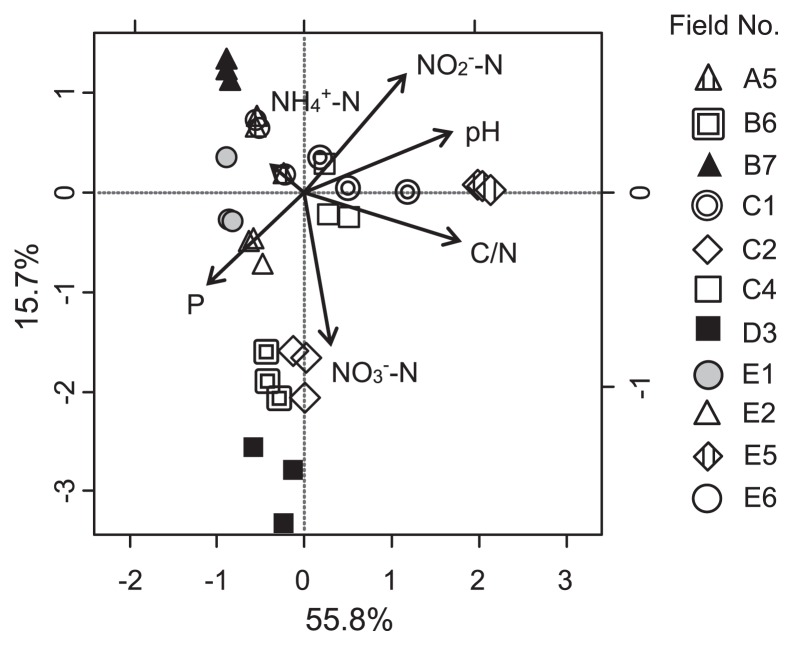
CCA ordination plots for the *Burkholderia* community in the sugarcane field soils. The percentage of variation explained by each axis is shown on the axes. CCA was performed in the R programming ver. 15.2.1 using vegan packages.
