# Supplementary material for: A Fine-Scale Phylogenetic Analysis of Free-Living Burkholderia Species in Sugarcane Field Soil
Source: Microbes Environ. 2014 Nov 20;29(4):434–7. doi: 10.1264/jsme2.ME14122 (PMC4262370; doi:10.1264/jsme2.ME14122)
Supplement: Supplementary file 1 [file 29_434_s1.pdf]

Table S1 Chemical characteristics of soil samples investigated in this study

Field No.	pH	C/N	NH_4^+-N (μg/g dry soil)	NO_2^--N (μg/g dry soil)	NO_3^--N (μg/g dry soil)	P (μg/g dry soil)
A5	4.64	8.6	14.4	0.13	6.1	56.3
B6	4.17	9.3	188.5	0.04	58.8	96.1
B7	5.10	6.5	303.8	0.15	11.5	69.5
C1	6.36	8.9	7.4	0.08	0.2	58.9
C2	5.31	8.6	83.1	0.10	81.2	77.8
C4	5.78	9.0	24.2	0.12	6.1	48.8
D3	4.79	9.3	32.7	0.08	50.3	79.5
E1	4.88	8.1	18.6	0.07	17.9	62.6
E2	5.20	5.9	352.4	0.10	68.5	118.6
E5	7.62	11.8	78.0	0.17	30.8	45.3
E6	6.08	7.9	39.4	0.12	9.7	61.7

All values were excerpted from a report by Tago *et al*. (doi:10.1264/jsme2.ME14124)

Table S2. Strains tested in PCR using *Burkholderia* specific primers Bf and Br

Strains	GenBank accession number
Burkholderia plantarii LMG9035^T	U96933
Burkholderia glathei ATCC29195^T	Y17052
Burkholderia kururiensis KP23^T	AB024310
Burkholderia sordidicola S5-B	AF512826
Burkholderia caledonica LMG19076	AF215704
Burkholderia fungorum LMG16225	AF215705
Burkholderia multivorans LMG13010^T	Y18703
Burkholderia ferrariae NBRC106233^T	AB537487
Burkholderia silvatlantica NBRC106337	AB537488
Burkholderia mimosarum PAS44	AY752958
Cupriavidus gilardii LMG5886	AF076645
Cupriavidus metallidurans CH34	Y10824
Cupriavidus necator JCM 11282	AB594764
Cupriavidus oxalaticus DSM1105	AF155567
Cupriavidus pauculus CIP105943	EU024165
Pandoraea norimbergensis LMG13019	AF139171
Ralstonia pickettii ATCC27511	AY741342
Ralstonia mannitolilytica LMG6866	AJ270258
Wautersia numazuensis TE26	AB104447

Table S3 Relative distribution of sequence reads obtained using the *Burkholderia* specific primers on sugarcane field soils.

Genus	Relative abundance (%)*
Burkholderia	95.8
Azohydromonas	1.1
Caldimonas	0.4
Rubrivivax	0.2
Roseateles	0.2
Aquabacterium	0.2
Methylibium	0.1
Ideonella	0.1
Rhizobacter	0.1
Unclassified	1.7

*The genera representing > 0.1% of the total sequences are shown in this Table.

Table S4 Number of sequence reads and OTUs

Field No.	Sequence reads per field	Sequence reads per subsample	Number of OTUs				Coverage (%)	Chao1	Data ID*
			Total	SBE	PBE	BCC			
A5	26,621	7,300	58	21	36	1	100	58	4577587.3
		8,105	55	20	33	2	100	55	4577588.3
		11,216	55	20	35	0	100	55	4577589.3
B6	18,421	10,126	51	22	27	2	100	51	4577590.3
		7,379	47	20	26	1	100	47	4577591.3
		916	29	12	17	0	100	29	4577592.3
B7	36,277	4,655	31	11	20	0	100	31	4577593.3
		13,429	35	12	22	1	100	35	4577594.3
		18,193	41	16	25	0	100	41	4577595.3
C1	16,007	4,306	22	12	10	0	100	22	4577596.3
		10,778	45	22	22	1	100	45	4577597.3
		923	27	14	13	0	100	27	4577598.3
C2	12,250	3,608	39	12	26	1	100	39	4577599.3
		3,337	46	16	28	2	100	46	4577600.3
		5,305	53	19	33	1	100	53	4577601.3
C4	25,474	9,343	52	19	32	1	100	52	4577602.3
		4,207	46	19	27	0	100	46	4577603.3
		11,924	55	18	37	0	100	55	4577604.3
D3	25,829	10,115	62	23	35	4	100	62	4577605.3
		10,156	64	22	39	3	100	64	4577606.3
		5,558	54	20	32	2	100	54	4577607.3
E1	26,263	6,771	43	16	27	0	100	43	4577608.3
		11,010	39	16	22	1	100	39	4577609.3
		8,482	43	17	24	2	100	43	4577610.3
E2	15,809	3,656	41	15	22	4	100	41	4577611.3
		7,455	49	18	30	1	100	49	4577612.3
		4,698	49	17	29	3	100	49	4577613.3
E5	73,861	31,725	42	14	25	3	100	42	4577614.3
		22,543	42	16	22	4	100	42	4577615.3
		19,593	42	14	23	5	100	42	4577616.3
E6	88,909	34,323	70	24	43	3	100	70	4577617.3
		32,393	69	22	43	4	100	69	4577618.3
		22,193	68	22	42	4	100	68	4577619.3
Total		365,721	95	29	58	8			

* The nucleotide sequences have been deposited in the MG-RAST database (http://metagenomics.anl.gov/) as the "*Burkholderia* in the sugarcane fields on the Minami-Daito island" project under the ID: 4577487.3-4577619.3.

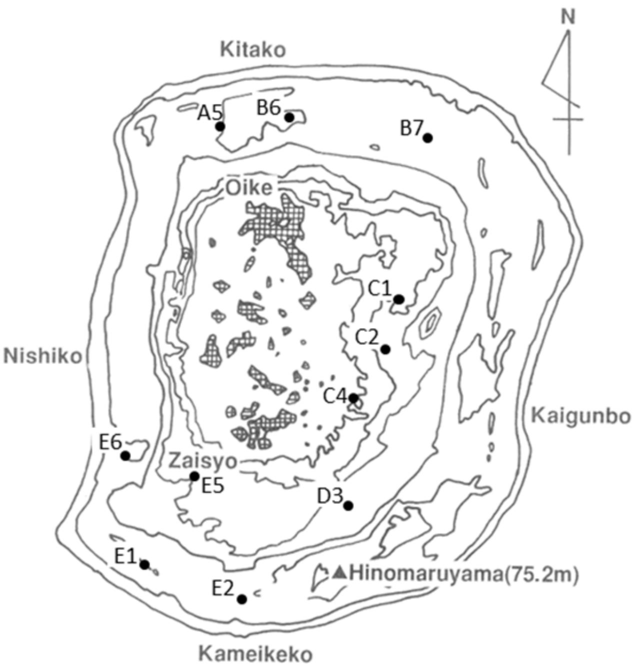

Fig. S1 Map of Minami-Daito Island. Field numbers are indicated.

Contour map is provided by the Geospatial Information Authority of Japan.

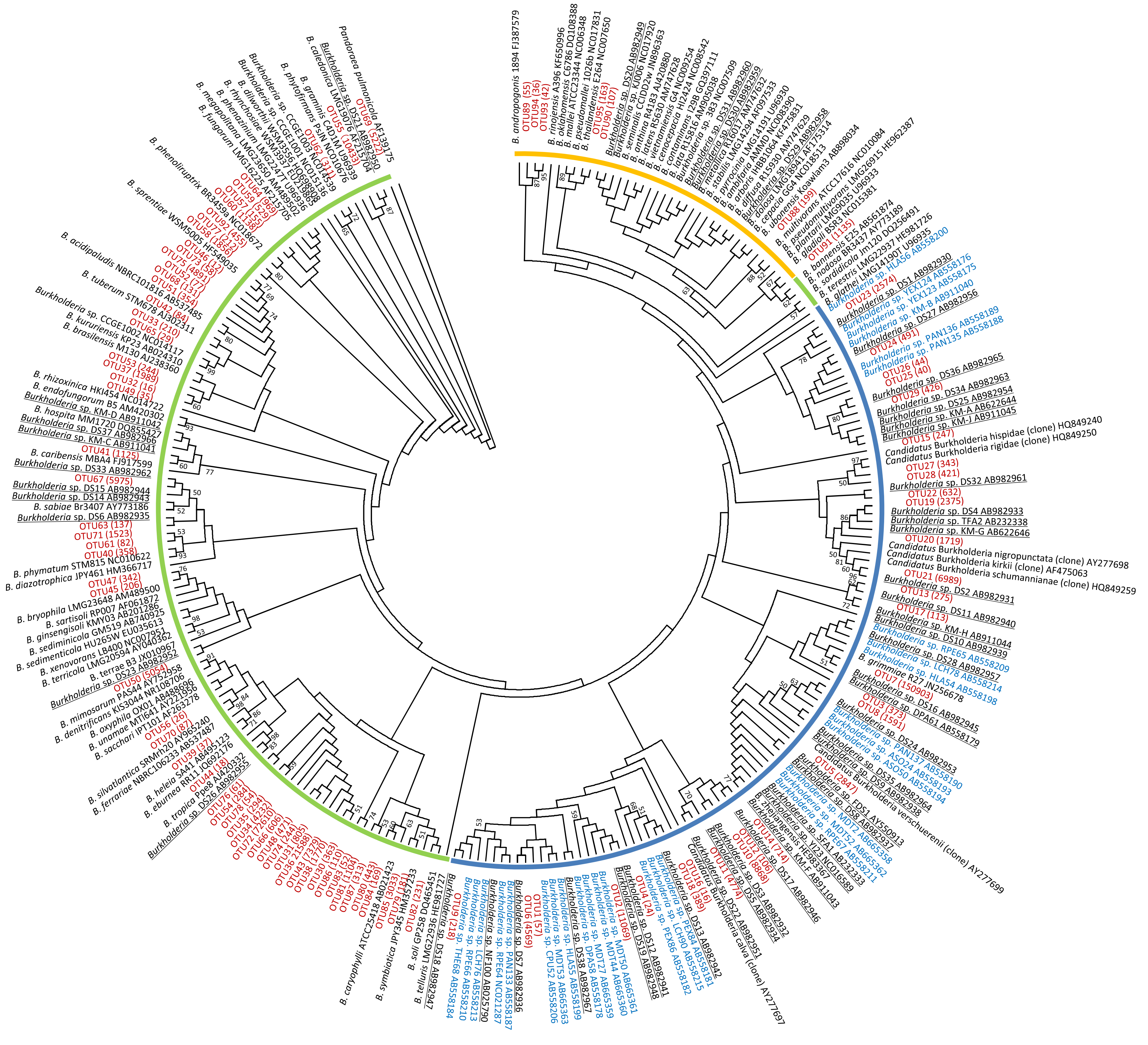

Fig. S2 Phylogenetic tree of *Burkholderia* based on partial 16S rRNA gene sequences, showing the positions of OTUs and representatives. Fenitrothion degraders are underlined. Several known symbionts obtained from stinkbugs were represented in blue. A circle between the tree and the sequence names indicate the division of the genus *Burkholderia* by (7): Blue, the SBE group; orange, BCC; green, the PBE group. Number of sequences in each OTU are given in parentheses followed by the OTU number. Accession numbers of reference sequences in the NCBI database are indicated. The 16S rRNA gene sequence of *Pandoraea pulmonicola* was used as an outgroup. Branch nodes with >50% supporting bootstrap values (1000 replicates) are shown.
